# Cutaneous Vascular Neoplasms of Uncertain Biological Behavior

**DOI:** 10.3390/biology10111160

**Published:** 2021-11-09

**Authors:** Kasey J. McCollum, Rami N. Al-Rohil

**Affiliations:** 1 Department of Pathology, Duke University Medical Center, Durham, NC 27710-0001, USA; kasey.mccollum@duke.edu; 2 Department of Dermatology, Duke University Medical Center, Durham, NC 27710-1000, USA

**Keywords:** intermediate potential, cutaneous neoplasm, hemangioendothelioma, cutaneous tumors, vascular neoplasm

## Abstract

**Simple Summary:**

Cutaneous vascular neoplasms of uncertain biological behavior present a diagnostic and therapeutic challenge to physicians. The rarity of these lesions and the recent recognition of such entities suggests an extensive amount of knowledge remains lacking concerning effective treatments, accurate prognosis, and follow-up recommendations for patients with superficial vascular tumors. The objective of this manuscript is to compile a comprehensive summary of the current literature on neoplasms limited to the skin and of vascular origin that are currently categorized as indeterminate biological potential. By compiling numerous studies and summarizing the aggregate findings, this paper aims to offer providers a condensed yet detailed report of the entirety of what is known regarding these lesions. The information will aid physicians in the process of diagnosing, treating, prognosticating, and following up these rare tumors.

**Abstract:**

Neoplasms of uncertain biological behavior present physicians with a genuine conundrum in practice. Cutaneous vascular neoplasms within this category are exceedingly rare, possessing significant gaps and uncertainty in many facets of clinical practice. Firstly, lesions were selected for review based on their categorization as indeterminate behavior, indicating the potential for local recurrence and rarely metastasize. After identification of the target lesions, a comprehensive review of the literature using national databases produced several landmark studies and case series regarding these neoplasms. Limiting the review to only cutaneous limited tumors narrowed the pool of studies; however, quite a large sum of papers remained. Examination of each paper yielded beneficial results on diagnosing, effective treatments, follow-up findings, and prognosis for each indeterminate lesion discussed. Overall, the literature search combined the molecular, histologic, immunohistochemical, surgical strategies to develop an up-to-date and comprehensive framework to guide physicians when encountering such lesions. The tumors reviewed include: kaposiform hemangioendothelioma, endovascular papillary angioendothelioma, pseudomyogenic hemangioendothelioma, retiform hemangioendothelioma, epithelioid hemangioendothelioma, and composite hemangioendothelioma.

## 1. Introduction

Recently the field of cutaneous vascular neoplasms has recognized a significant number of unique and challenging entities [1,2,3,4,5,6,7,8,9]. While tumors have been classified into benign or malignant categories, others remain poorly characterized and thus are referred to as „cutaneous neoplasms of uncertain biological potential” [10,11,12]. These cases, while not the most common in daily practice, present quite a diagnostic and prognostic conundrum for both dermatopathologists and treating clinicians. The recent explosion of innovative molecular technologies has allowed for more accurate classification of these lesions while allowing scientists to investigate the clinical implications of the associated genomic and epigenetic alterations [3,5,13]. This manuscript discusses the current literature available and provides much-needed insight for classification, diagnosing, and treating patients with these rare cutaneous vascular neoplasms. Such an overview aims to both stimulate the emergence of an expert opinion on strategies to adopt in the future to promote the development of innovative diagnostic, prognostic, and therapeutic modalities for these grey-zone entities.

## 2. Materials and Methods

Original publications considered for discussion were found using the PubMed database. Searches used keywords including “cutaneous tumor,” “vascular lesion,” “uncertain biological behavior,” as well as combinations of all the above terms. Neoplasms to be discussed were selected based upon their current classification as neither benign nor malignant but labeled as indeterminate. Articles selected for review mainly were written within the last two decades and were accepted to peer-reviewed journals. Case reports, case series, and randomized controlled trials were all included in the inclusion criteria. Full-text papers were retrieved through Duke University Libraries access.

Excluded papers mainly discussed variants of vascular tumors that were deep-seated soft tissue variants and were not considered cutaneous in nature. Excluded articles included those that were not written in the past twenty years (except for historical aspects papers) and papers that were not evaluated by a peer-review process.

## 3. Results and Discussion

### 3.1. Kaposiform Hemangioendothelioma 

#### 3.1.1. Historical Aspects

Kaposiform hemangioendothelioma (KHE) is an uncommon and indeterminate tumor of endothelial origin typically presenting in childhood, which was first described in 1993 by Zuckerberg and colleagues [1,10]. Before its description by Zuckerberg et al., similar tumors were reported under a variety of other names, including Kaposi-like hemangioma, hemangioma with Kaposi-like features, Kaposi-like infantile hemangioendothelioma, congenital hemangioendothelioma [2,10]. Earlier cases reported had similar histologic features consisting of a multinodular infiltrative process and complex vascular proliferation. These lesions showed varying degrees of overlap with capillary hemangioma and Kaposi sarcoma (KS). Additionally, Zukerberg et al. pointed out the association between KHE, Kasabach-Merrit phenomenon (KMP), and lymphatic abnormalities. Currently, more than 200 cases have been described in the literature [3,4,5].

#### 3.1.2. Epidemiology and Demographics

Most tumors present in infancy and childhood and are relatively uncommon in adults. Furthermore, up to 70% of reported cases of Kaposiform hemangioendothelioma cases present within the first year of life [4]. No sex predilection has been identified, equally affecting males and females [3,5].

#### 3.1.3. Clinical Presentation 

**Figure 1:**
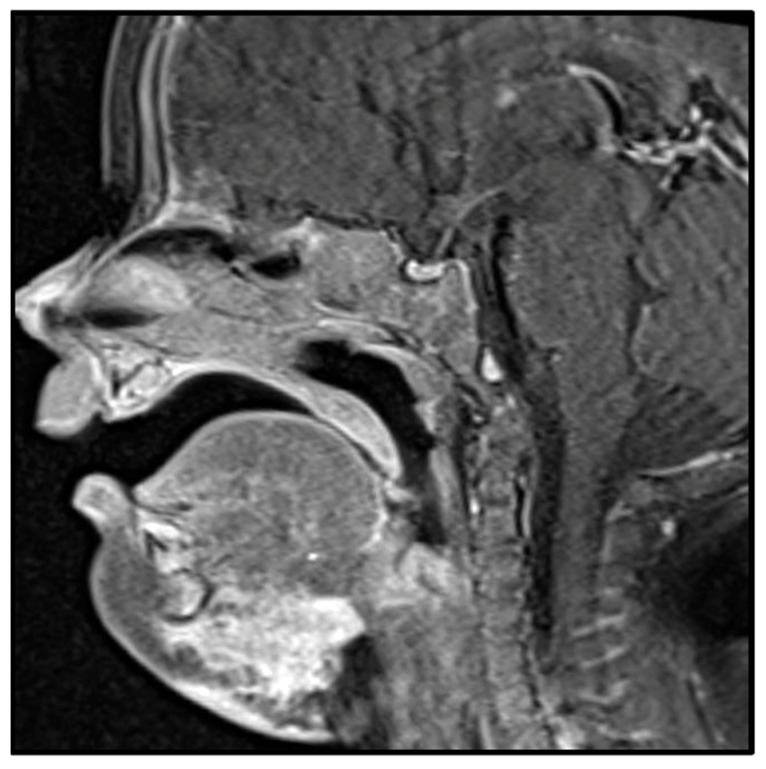
MRI image of kaposiform hemangioendothelioma involving the submental region with subcutaneous and soft tissue involvement.

Most cutaneous lesions present as ill-defined erythematous, or violaceous plaques, which may or may not be painful. Lesions most commonly develop on the extremities/joints, chest/abdominal wall and are less likely to be on the head and neck (Figure 1) [6,7,8,9]. Clinically, these lesions can be associated with KMP, which consists of consumptive coagulopathy and thrombocytopenia. The highest risk for coagulopathy is generally associated with retroperitoneal and intrathoracic tumors but is also associated with large cutaneous lesions [5].

#### 3.1.4. Histologic Features

**Figure 2:**
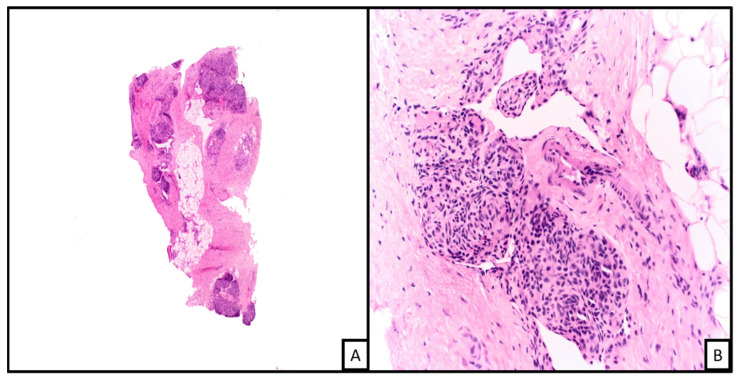
An example of kaposiform hemangioendothelioma. Histopathologic examination on low power demonstrates a vascular proliferation involving connective and subcutaneous tissue in a lobular configuration ((**A**), H&E 2×; on higher magnification the nodules are formed of spindled shaped endothelial cells with cleft-like spaces at the periphery ((**B**), H&E 20×).

Histologically, cutaneous forms demonstrate nodules of irregular tumor cells infiltrating the dermis comprised of spindle-shaped endothelial cells and ectatic slit-like vasculature channels at the periphery. Occasionally, the spindled tumor cells can demonstrate a swirling growth pattern, invoking a somewhat glomeruloid appearance. Tumor cells have uniform hyperchromatic spindled nuclei arranged in short fascicles with slit-like vascular lumina surrounded by a dense collagenous stroma (Figure 2) [6,7,8,9,10,11]. Additionally, most cases show extravasated red blood cells, single cells with lumina containing RBCs, dilated lymphatic vessels, eosinophilic bodies, and hemosiderin amidst vascular cells and fibrin thrombi. Rare mitoses, mild nuclear variation with minimal atypia, and no necrosis are all characteristics of KHE [9].

#### 3.1.5. Ancillary Testing and Immunohistochemical Stains

Immunohistochemistry demonstrates positivity for CD31, CD34, FLI-1, EGR, and podoplanin (D2-40), as well as being negative for GLUT1 and HHV8 [2,5,9].

#### 3.1.6. Genetics and Molecular

*Prox1* has been reported as an important gene related to the aggressive and infiltrative behavior of these vascular neoplasms in mouse models. However, this gene has yet to be identified in human tumor tissues [5]. Mutations in *GNA14* (a G-coupled protein receptor gene) have been reported in up to 33% of KHE specimens which affects the mitogen activating pathway kinase-extracellular signal-regulated kinase pathway (MEK) [3]. The identification of these mutations is revolutionizing the way in which lesions are diagnosed and understood. Mutations and their associated pathways are offering potential targets for the development of innovative treatments which may improve prognosis.

#### 3.1.7. Challenges to Classification and Differential Diagnosis

Considering the histologic appearance and the age of presentation, KHE may be difficult to differentiate from other cutaneous vascular lesions, including Kaposi Sarcoma, tufted angioma, and juvenile hemangioma. IHC stains are exceedingly helpful in confirming a definitive diagnosis. Juvenile hemangioma tends to show a more superficial growth pattern but rarely can show a deep infiltrative growth similar to KHE; however, immunohistochemically, KHE is consistently negative for GLUT1, which is strongly expressed in juvenile hemangioma, aiding in differentiating these two entities. Kaposi sarcoma is more commonly seen in older age groups and can occur in the setting of immunocompromised patients (e.g., iatrogenic and AIDS). Kaposi sarcoma immunohistochemically shows nuclear HHV8 transcripts, which is negative in KHE lesions. Probably the most challenging to differentiate is the Tufted angioma from the KHE. Histologically and immunophenotypically, the lesions overlap several characteristic features leaving only the growth pattern and IHC to aid in distinction. Tufted angiomas are benign tumors mainly restricted to the dermis, while KHE often shows an infiltrative growth pattern into the subcutaneous tissue. A monoclonal antibody marker for lymphatic endothelium proves useful for differentiating KHE and TA. KHE shows peripheral podoplanin (D2-40) positivity in proliferating capillaries and is unreactive primarily in the surrounding dilated vessels. In contrast, tufted angioma shows podoplanin (D2-40) partial positivity in surrounding dilated vessels and is negative in the proliferative capillaries [1]. 

#### 3.1.8. Current Literature on Biological Behavior and Treatment

KHE has been classified as a borderline/indeterminate malignancy due to its infiltrative growth pattern, secondary associated systemic characteristics (KMP), and absence of spontaneous regression/self-healing [4]. The lesions seldom show distant metastasis and rarely metastasize to regional lymph nodes. Studies suggest that the trapping of RBCs and platelets within the lesion may underpin the localized intravascular coagulation (LIC) associated with KHE; however, more research is required to confirm this hypothesis. The LIC associated with KMP is one of the main reasons KHE is classified as an indeterminate tumor [10]. The severity of KMP increases with tumor size, retroperitoneal location, and deeper muscle infiltration, which may predict the clinical outcome for the patient. Mortality associated with KHE is currently reported at about 10% and is mainly related to the coagulopathy caused by KMP [4,6,8]. Based on these clinical and morphological factors, physicians can better determine the prognosis for their patients. First-line treatment for cutaneous lesions is generally regarded as a wide resection approach, in which all patients were cured without reoccurrence. For patients in which resection is not an option (depth of tumor/size of the tumor), treatment with vincristine, interferon-alpha, sirolimus has all proven to be effective in reducing the size of lesions. Certain studies reported that in patients treated with systemic therapies, regression occurred in 28% of patients receiving steroids, 39% with vincristine, 43% with interferon-alpha, 61% with anti-platelet agents. Most promising was the use of sirolimus-containing therapies, which found 100% regression/reduction in tumor size [6]. Additionally, a recent study conducted by Brill et al. demonstrated the utilization of transarterial embolization in combination with systemic sirolimus therapy in non-surgical candidates with KMP. The use of both therapies together showed rapid resolution of life-threatening KMP, engendering improved survival for patients with KHE [11].

### 3.2. Endovascular Papillary Angioendothelioma (EPA, Dabska Tumor)

#### 3.2.1. Historical Aspects

The first description of the Dabska tumor was recorded in 1969 by Maria Dąbska in a case report series of six children with various cutaneous lesions. Histology showed anastomosing vascular channels with atypical endothelial cells projecting into the lumen [12,13]. The entity was initially thought to be a low-grade angiosarcoma. Most of the cases in Dr. Dąbska’s case series were treated with radiation therapy and wide local excision. Other similar cases were reported by Phallen, Henschen, and Masson under different names [12]. Since its original description by Dabska, only around forty cases have been recorded [14].

#### 3.2.2. Epidemiology and Demographics

EPA tumors are particularly rare and often present in children (75%), with only a minority occurring in adults. Overall, studies have reported no clear gender preponderance [12,13,15]. 

#### 3.2.3. Clinical Presentation 

The lesions appear as an asymptomatic solitary plaque-like area affecting the skin and superficial soft tissue with overlying violaceous skin discoloration. The lesions can become quite large, with the average recorded size being about 2–3 cm in diameter [16]. The distal extremities are the most common site of occurrence but may arise in other locations like the head and neck, and trunk [13].

#### 3.2.4. Histologic Features

**Figure 3:**
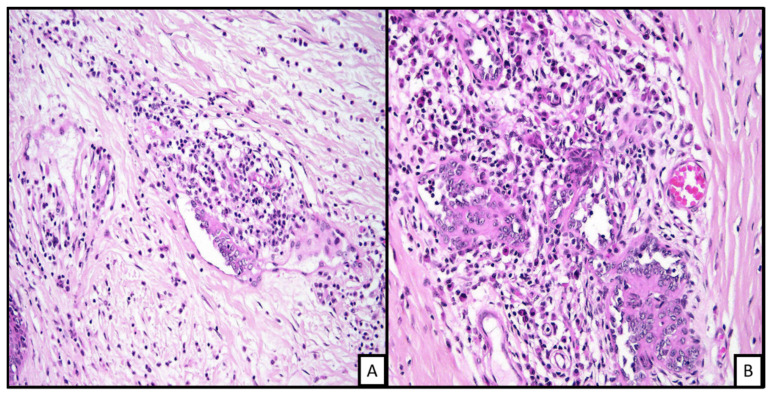
Histopathologic examination of endovascular papillary angioendothelioma shows a vascular proliferation in the dermis. The dilated vascular channels show some surrounding mixed inflammatory infiltrate with some collagen fibrosis. The endothelila lining shows some hobnailing with plump lining ((**A**), H&E 20×; (**B**), H&E 40×).

Microscopically, the lesions show a dermal proliferation of vessels lined by enlarged cuboidal endothelial cells. Intravascular papillary projections with hyaline cores and the endothelial cells show prominent hobnail features with plump, rounded profiles protruding into the lumina (Figure 3). In some cases, there may be cytoplasmic vacuolation, and mitotic figures are usually absent. Surrounding the vessels is usually a lymphoid infiltrate and sclerotic collagen. The vessels often extend into the subcutaneous tissues [12,13,14]. 

#### 3.2.5. Ancillary Testing and Immunohistochemical Stains

EPAs show positivity for vascular markers as CD31, CD34, ERG, and FLI-1. Most case reports have documented positivity for lymphatic markers D2-40 and VEGFR-3 [14,15].

#### 3.2.6. Genetics and Molecular

Currently, there are no known genetic or molecular abnormalities associated with EPAs as they are thought to arise from a preexisting vascular malformation or in an area of chronic lymphedema [16].

#### 3.2.7. Challenges to Classification and Differential Diagnosis

The top differential diagnoses include retiform hemangioendothelioma, composite hemangioendothelioma, and Kaposi sarcoma. In the case of retiform hemangioendothelioma, the presentation age is helpful because most occur in older adults. Retiform hemangioendothelioma shows significant overlap with EPA histopathologically, including the papillary structures. These two entities are thought to belong on the same spectrum of tumors [16]. Composite hemangioendothelioma are composed of at least two distinct hemangioendotheliomas (discussed in Section 3.6). Kaposi sarcoma is positive by HHV8 immunohistochemistry and lacks the papillary structure typical of EPA [15].

#### 3.2.8. Current Literature on Biological Behavior and Treatment

EPA is as indeterminate/borderline tumors because of their potential for local reoccurrence and low-grade metastasis [16]. Due to its potential for nodal and distant metastasis, a complete physical and radiographic workup is recommended. Currently, the recommended treatment is complete wide local surgical excision to prevent metastasis. The rarity of the tumors has made it difficult to evaluate the benefit of radiation therapy in a randomized trial. The current literature suggests the use of radiation in tumors with high-risk features, including size >5 cm, deep invasion, and positive margins on resection [15,16]. However, local recurrence is quite common. Due to the rarity, there is not yet a standard protocol for follow-up after resection. The NCCN Practice Guidelines for Soft Tissue Sarcoma recommend chest imaging every 3 to 6 months for 2–3 years following resection, then every six months for another two years [17].

### 3.3. Pseudomyogenic Hemangioendothelimoa (PMH)

#### 3.3.1. Historical Aspects

Mirra et al. first described five cases of a unique multifocal soft tissue tumor showing spindled cells positive for vimentin and keratin, leading to its original name of a fibroma-like variant of epithelioid sarcoma [18,19]. In 2003, Billings et al. described several cases of vascular tumors that appeared to be low-grade and recommended renaming the entity to „epithelioid sarcoma-like hemangioendothelioma” [18,20]. The work of Hornick and Fletcher culminating in a case series of fifty patients lead to the diagnostic term pseudomyogenic hemangioendothelioma [21]. Currently, the World Health Organization places PMH under the category of rarely metastasizing vascular neoplasms of intermediate malignant potential [22].

#### 3.3.2. Epidemiology and Demographics

PMH is quite rare, generally appearing in young adults with a mean average of presentation being 30 years old. PMH is uncommonly found in people over 40 years old. Studies have shown a male predilection, up to a ratio of 7:1 [18,19].

#### 3.3.3. Clinical Presentation 

The most common location is in the lower extremity; however, other locations have been reported, including the upper extremity, trunk, and head and neck. They can present as a painful or painless nodule and can be multicentric in up to 50% of patients. The cutaneous form of this tumor may show ulceration. The tumors are commonly poorly demarcated and are often less than 3 cm in size [21,22].

#### 3.3.4. Histologic Features

**Figure 4:**
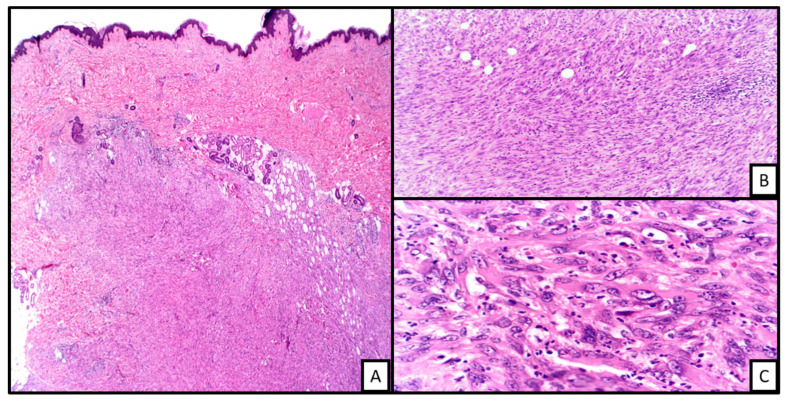
An example of pseudomyogenic hemangioendothelioma. On low power, there is a spindle cell proliferation in the deep dermis ((**A**), H&E 2×). On closer inspection, the cells are in the form of fascilces and sheets with prominent Intratumoral neutrophilic infiltrate ((**B**), H&E 20×). The cells show rhabdomyoblast-like morphology ((**C**), H&E 40×).

Histologically PMH shows infiltrative borders with ill-defined nodules, sheets, and fascicles of plump spindled and epithelioid cells with abundant eosinophilic cytoplasm. The tumor cell nuclei are round with vesicular chromatin and have variable nucleoli. Often there is mild to moderate atypia, with marked atypia exceedingly rare [18,19,20,21]. Occasionally tumors can show intracytoplasmic lumen formation focally. Mitoses are often scant. Some cells resemble rhabdomyoblasts showing large eccentric nuclei with eosinophilic cytoplasm but are not indeed rhabdomyoblasts and do not show rhabdomyoblastic differentiation immunohistochemically (thus the term pseudomyogenic) (Figure 4). The tumor cells are often surrounded by a fibrous or desmoplastic stroma, with a myxoid background being much less likely [18]. There is often a vigorous neutrophilic infiltrate and an absence of well-formed vascular elements. This latter feature makes intralesional hemorrhage uncommon. Small foci of necrosis or vascular invasion have been reported in some tumors [21,22,23,24].

#### 3.3.5. Ancillary Testing and Immunohistochemical Stains

Characteristic IHC stains show positivity for cytokeratin AE1/AE3, ERG, FLI-1 and are often negative for other keratins. CD31 is reported to be positive in about 50% of cases, along with retained nuclear INI1 and positivity for FOSB. They are consistently negative for CD34. Some studies have found that there can be focal SMA positivity and up to 30% of cases. Negative stains include EMA, S100, desmin, myogenin, and CAMTA1 [18,19,20,21,22,23,24].

#### 3.3.6. Genetics and Molecular

There is a characteristic translocation occurring in these tumors, which is the t(7, 19) (q22;q13), resulting in the *SERPINE1-FOSB* fusion gene product. However, this translocation has not been described in all cases of PMH, yet it is the only identified translocation to date [18]. SERPINE1 is also referred to as plasminogen activator inhibitor type 1 (PA-1), which encodes for the serine proteinase inhibitor family and inhibits fibrinolysis [23]. FOSB regulates induced expression of the extracellular matrix proteins within smooth muscle [24]. The *SERPINE1-FOSB* gene fusion leads to overexpression of the *FOSB* gene, which is thought to drive the growth and development of PMH [23,25]. These gene alterations may provide valuable targets for pharmacotherapy development in the future.

#### 3.3.7. Challenges to Classification and Differential Diagnosis

The differential diagnosis for a PMH includes an epithelioid sarcoma, sarcomatoid carcinoma, and an epithelioid hemangioendothelioma. An epithelioid sarcoma shows a dominant nodular growth pattern and often shows palisading necrosis. Epithelioid sarcoma also shows a loss of INI-1 to confirm the distinction. Immunohistochemically, epithelioid sarcomas tend to express pan-cytokeratin markers and CD34, even sometimes ERG and FLI-1; however, CD31 is usually negative. Sarcomatoid carcinoma usually occurs in older age groups in the skin and shows more pronounced cytologic atypia and positivity for additional keratin markers but negative for vascular markers such as CD31 and CD34. Epithelioid hemangioendothelioma has more cords and small fragments of the small epithelioid cells with intracytoplasmic lumina, which is uncommon in the PMH. Epithelioid hemangioendothelioma generally shows a myxohyaline stroma in contrast to the fibrous stroma of PMH. For complete distinction, epithelioid hemangioendothelioma is known for its characteristic translocation t(1;3) with the *WWTR1-CAMTA1* fusion gene. PMH also may be confused with a fibrous histiocytoma/dermatofibroma; however, fibrous histiocytoma lacks the plump myoid-appearing tumor cells and abundant neutrophilic infiltrate and would demonstrate collagen trapping noted mostly at the periphery of these tumors [18,24,26].

#### 3.3.8. Current Literature on Biological Behavior 

Due to the nature of local reoccurrence and potential for metastasis, the PMH is currently classified as a borderline entity. Presently complete surgical excision is the first-line recommended treatment [18,19,26]. There is no known role for adjuvant therapy in the literature. These tumors often show a very indolent clinical course; however, local reoccurrence after resection is quite common. Reoccurrence can show multiple lesions occurring over a long period of time, with true metastasis being exceedingly rare. The most reported sites of true metastasis include the bone, lymph nodes, lung, and scalp. The multi-centricity of reoccurrences is often mistaken for local-regional metastasis. PET scans at the time of diagnosis can be used to determine distant metastasis due to the extreme PET avidity of soft tissue and bone metastasis [20,21,22,23,24]. A promising study showed the effectiveness of telatinib (blocks VEGF signaling pathway) as a therapy for PMH. The study demonstrated that the drug indirectly interferes with the self-regulated expression of the fusion gene product leading to complete clinical remission in their patient. This development demonstrates a very promising treatment option for patients who present with multifocal and unresectable PMH [25].

### 3.4. Retiform Hemangioendothelimoa (RHE)

#### 3.4.1. Historical Aspects

RHE was first described in 1994 by Calonje et al. when it was distinguished as a separate entity from cutaneous angiosarcoma in a series of fifteen cases [27,28]. In 2010, Hirsh et al. presented a case series of another 25 cases which fit the morphology of the RHE, and since then, there have been a few more cases reported, making the RHE overall exceedingly rare tumor [28,29,30,31].

#### 3.4.2. Epidemiology and Demographics

RHE is a rare tumor that may be associated with underlying lymphedema or preceding radiation therapy. It occurs most often in middle-aged adults, but some cases have been reported in children [27]. The preponderance of tumors occurs in females [27,28,29,30,31].This tumor typically occurs on the trunk or extremities and is locally aggressive and rarely metastasizes; however, several cases reported lymph node metastases [32,33,34,35,36,37,38]. 

#### 3.4.3. Clinical Presentation 

RHE presents as a large nodular or plaque-like lesion on the trunk or extremities. Usually poorly circumscribed and can be either subcutaneous or dermal based. RHE can attain a large size, up to 12 cm, and are often asymptomatic and slow-growing [28,30].

#### 3.4.4. Histologic Features

**Figure 5:**
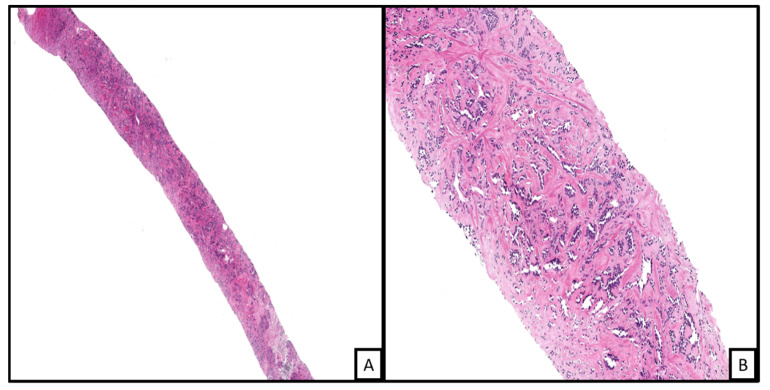
Needle core biopsy of a retiform hemangiendothelioma. The vascular proliferation is in the form of arborizing blood vessels in a sclerotic stroma ((**A**), H&E 4×). Higher power examination shows the prominent slit-like spaces with plump endothelial lining ((**B**), H&E 20×).

Microscopically, RHE shows very distinctive elongated, arborizing blood vessels that resemble the rete testis, giving the tumor its namesake. There are often papillary-like intraluminal projections and vascular spaces lined by hobnailed endothelial cells with oval nuclei and mild hyperchromasia (Figure 5) [29,31]. Histologically, as discussed in a previous section, RHE shows significant overlap with the Dabska tumor [17,28,33].

#### 3.4.5. Ancillary Testing and Immunohistochemical Stains

Tumor cells are reactive for vascular endothelial markers, including CD31, CD34, ERG, and FLI-1. Most reported cases are negative for lymphatic markers like D2-40 and VEGFR-3; however, a few rare cases have been identified expressing lymphatic markers [27,30,31,34].

#### 3.4.6. Genetics and Molecular

To date, no significant molecular abnormalities have been identified in RHEs.

#### 3.4.7. Challenges to Classification and Differential Diagnosis

Endovascular Papillary Angioendothelioma (EPA, Dabska tumor), composite hemangioendothelioma (HE), and Kaposi Sarcoma are all on the differential when presented with this classic histology. EPA may be the hardest to distinguish from RHE, leaving the age of the patient to guide the diagnosis, with EPA being much more common in children and RHE occurring in middle-aged adults. EPAs typically have more papillary structures and lack prominent rete-style vessels. Additionally, EPAs are positive for lymphatic markers D2-40 and VEGFR-3, which are primarily negative in RHE, but that immunophenotype is not a reliable distinguisher [17,28]. A composite hemangioendothelioma contains at least two different distinct areas of hemangioendotheliomas. For differentiating Kaposi Sarcoma, a clinical history of immunosuppression or older age group is always helpful. The lack of a rete-like pattern and a positive for HHV8 will distinguish a diagnosis of Kaposi sarcoma. However, a case report of an RHE showed immunoreactivity for HHV8, leaving other morphological features to aid in distinguishing the two entities [32]. Another critical distinction is to distinguish an RHE from cutaneous angiosarcoma due to treatment implications and the malignant potential of cutaneous angiosarcomas. Clinically, cutaneous angiosarcomas tend to occur in older populations and demonstrate higher levels of cytologic atypia and high mitotic rate [27,31].

#### 3.4.8. Current Literature on Biological Behavior 

Complete wide surgical excision is the current recommended protocol [28]. RHE is locally aggressive, yet metastases are rarely reported [30,31,33,36]. Due to the high rate of recurrences, it remains essential for tumor-free margins and close long-term follow-up. One theory as to why RHE has a high recurrence rate is since the tumor has a dissecting growth pattern and remains difficult to negative margins [35]. For cases that are not amenable to resection (i.e., facial location), a promising study demonstrated that definitive treatment with chemoradiation comprised of low-dose cisplatin and radiotherapy was successful in achieving regression [29].

### 3.5. Epitheloid Hemangioendothelioma (EHE)

#### 3.5.1. Historical Aspects

Epithelioid hemangioendothelioma’s were first described by Deil and Liebow in 1975 in a case series of twenty patients with mostly soft tissue tumors and a few superficial tumors [37,38,39]. Weiss coined the term EHE when she published a case series in 1982 detailing forty-one cases, with the majority comprising soft tissue tumors and only a few cutaneous variants. Indeed, cutaneous EHEs prove to be exceedingly rare, with only around 22 case reports in the literature [40,41,42].

#### 3.5.2. Epidemiology and Demographics

This extremely rare tumor is most common in people aged 30 to 50 and is uncommon in children [39,40,43]. Studies have indicated a slight female predominance [38,42].

#### 3.5.3. Clinical Presentation 

Cutaneous EHE is even rarer than their soft tissue counterpart and is found in wide distribution, including the extremities and head and neck region [41,44,45,46]. EHE has been reported to present as a solitary painful mass, verrucous mass, erythematous mass, multiple dome-shaped nodules and can be partially indurated or ulcerated. Due to its presentation, it can often be misdiagnosed as a scar or infection [39,40,41,42].

#### 3.5.4. Histologic Features

**Figure 6:**
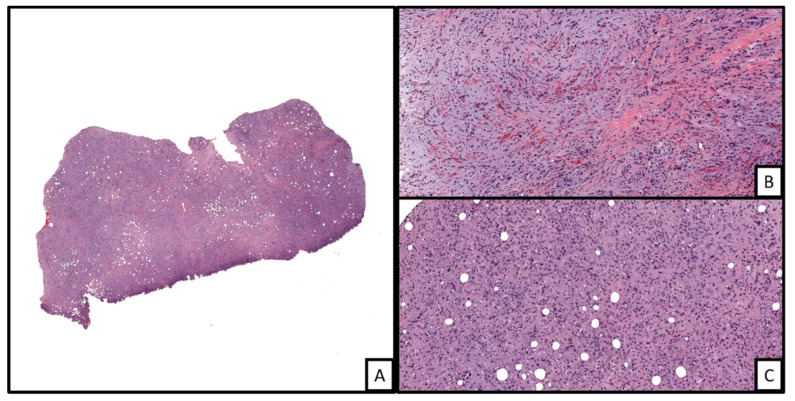
An example of epithelioid hemangioendothelioma involving the dermis ((**A**), H&E, 2×). The tumor is markedly cellular with some areas of myxohyaline matrix deposition in the background ((**B**), H&E 20×). In other areas, the tumor shows sheets of cells displaying distinct cells borders with neovascularized lumina formation with some containing red blood cells (i.e., blister cells) ((**C**), H&E 20×).

EHE is thought to arise from preexisting vessels and is comprised of bland-appearing epithelioid endothelial cells with intracytoplasmic vacuoles and a characteristic myxohyaline stroma. Tumor cells demonstrate an infiltrative growth pattern without any defined lobular structure [39,41]. The epithelioid cells can be arranged in cords, single cells, or in small aggregates. The cells are usually pale or have densely eosinophilic cytoplasm with intracytoplasmic vacuoles representing primitive vascular lumina, also known as blister cells. These vacuoles may contain erythrocytes. The nuclei are often small and vesicular and may or may not have small nucleoli present. Occasional tumor cells can show marked nuclear pleomorphism and hyperchromasia, but the mitotic rate is often low (less than three mitotic figures per 50 high power fields) [39,40,44,45,47]. The characteristic myxoid to hyaline stroma contains sulfated acid mucopolysaccharides. Typically, well-formed vascular channels are absent, although EHE can arise from large vessels, which can sometimes lead to the obliteration of the lumina (Figure 6). There also may be metaplastic calcification or ossification in a minority of cases, with necrosis being rare [39,40,41,44,46].

#### 3.5.5. Ancillary Testing and Immunohistochemical Stains

IHC stains show immunoreactivity for vascular markers such as CD31, CD34, ERG, FL I-1. Additionally, some tumors show positivity for nuclear TFE3. Keratin can be positive in up to 35% of cases [39,41,45]. Immunohistochemical staining with CAMTA1 is a valuable adjunct tool for a definitive diagnosis. 

#### 3.5.6. Genetics and Molecular

Molecular analysis of these rare tumors has shown characteristic translocations, mainly t(1;3) (p36;q25), resulting in the *WWRT1-CAMTA1* gene fusion product. Some cases have shown a *YAP1-TFE3* fusion gene. Tumors with this fusion product are often densely eosinophilic with more developed vascular formation [48]. It appears that although these characteristic translocations have been found in the majority of EHE, their clinical significance is uncertain [47,48,49,50]. It is not yet useful for targeted pharmacotherapy. The translocation can be utilized to help distinguish it from other histologically similar tumors.

#### 3.5.7. Challenges to Classification and Differential Diagnosis

The differential for EHE includes epithelioid hemangioma, epithelioid angiosarcoma, and epithelioid sarcoma. Epithelioid hemangioma generally shows a well-developed lobular architecture with well-formed vascular channels helping to distinguish it from EHE [42]. They also show a characteristic mixed chronic inflammatory infiltrate with prominent eosinophilia while lacking the genetic translocation characteristic of EHEs. Immunohistochemically epithelioid hemangioma has been shown to express FOSB. Epithelioid angiosarcoma usually occurs in the background of a traditional angiosarcoma; they exhibit large epithelioid tumor cells lining irregular vascular spaces or nests with mitosis being much more common, including atypical mitotic figures, which is rarely seen in typical EHE [47,51]. Additionally, in contrast to EHE, epithelioid angiosarcoma frequently shows tumor necrosis. Lastly, epithelioid sarcoma is most common in the distal extremities of younger patients. They often show a granuloma-like growth pattern with central necrosis and are negative for CD31 with the loss of INI-1 expression [40,41,42,44]. 

#### 3.5.8. Current Literature on Biological Behavior 

As with the other entities described previously, wide surgical excision with negative margins is the recommended treatment of choice for cutaneous EHE. There is no proven role for adjuvant chemotherapy or radiotherapy presently [43]. Overall, EHE shows a relatively indolent clinical course in most cases, with local recurrence being rare in cutaneous EHEs [41,42]. Tumors with high-risk features, including greater than 3 cm, necrosis, and greater than three mitoses per high-power field suggest a worse prognosis and often a significant decrease in the survival time of these patients [43]. Current literature suggests that the overall mortality rate is 10 to 20% in soft tissue-based tumors, but no deaths have been reported in relation to cutaneous EHEs [47,50]. Metastases are rare in cutaneous EHEs, occurring in about 5% of cases, with bone and lungs being the most common sites [40,41,44]. A recent study by Park et al. described a complete remission of a case using two stages of Mohs surgery on a posterior auricular lesion without evidence of recurrence in an 18-month follow-up [41]. Due to EHE’s infiltrative growth pattern and possible lymphatic involvement, careful follow-up is recommended, although no length of follow-up period has been defined in the literature [41,42,43,45,46].

### 3.6. Composite Hemangioendothelioma (CHE)

#### 3.6.1. Historical Aspects

Composite hemangioendothelioma is defined as an endothelial neoplasm of a low malignant potential composed of a mixture of histologically benign intermediate and malignant components [52]. It was first described in 2000 by Nayler et al., who reported a unique vascular tumor comprised of multiple histologic patterns of varying biological potentials [53,54]. Currently, a total of 38 cases have been recorded in the literature, making it a rare entity; however, their true incidence may be underrepresented given the histologic similarities to other more well-known lesions [54].

#### 3.6.2. Epidemiology and Demographics

CHE’s are exceedingly rare entities, most often occurring in the acral extremities, with the hands and feet being the most common site. They have been known to be associated with preexisting or congenital vascular malformations. Cases have mostly been reported in adults; however, a large age distribution has been reported in the literature ranging from infancy to young children and teenagers, up to the eighth decade of life [52,53,54]. In addition, some studies have reported a potential association of CHE’s with previous radiation therapy and longstanding lymphedema [55,56].

#### 3.6.3. Clinical Presentation 

Tumors typically present as enlarging nodular erythematous or violaceous mass on the hands or feet. They often have a red to purple color and maybe either nodular or multinodular with infiltrative borders. They range in size anywhere from 1 to 30 cm, with some cases showing satellite nodules, ulceration, bleeding, and edema. Foot and ankle lesions comprise about 50% of reported cases, and 25% occurring in the hand/forearm region. The oral mucosa is also a rare site for CHEs, making up about 12% of reported cases. Less common sites of involvement include the thigh, hip, back, scalp, and nose [52,53,54,55,57,58,59].

#### 3.6.4. Histologic Features

CHE is comprised of a complex mixture of a variety of vasiform patterns, including the previously discussed retiform hemangioendothelioma, which is the most common and usually dominant component of the tumor, as well as the epithelioid hemangioendothelioma pattern, which is the second most common pattern identified [53]. Other patterns may include well-differentiated angiosarcoma and the spindle cell hemangioma pattern. 

#### 3.6.5. Ancillary Testing and Immunohistochemical Stains

Immunohistochemical stains generally reflect the individual histologic pattern characteristics staining patterns. Often the tumor show variable expression for vascular endothelial markers, including CD31, CD34, ERG, FLI-1, ET-related gene, and von Willebrand factor [54,56,58,59].

#### 3.6.6. Genetics and Molecular

Molecular findings in composite hemangioendothelioma’s are consistent with the individual histologic patterns’ characteristic molecular findings. One study by Perry et al. found one case with *PTBP1-MAML2* translocation and one case with *EPC1-PHC2* fusion transcripts [60]. To date, the literature does not report any clinically significant and unique molecular or genetic findings in CHE.

#### 3.6.7. Challenges to Classification and Differential Diagnosis

As described in previous sections, the differential diagnosis for CHE includes the histologic patterns that comprise the tumor, including RHE, EHE, and well-differentiated angiosarcoma [59]. To help differentiate well-differentiated angiosarcoma, one would expect to see dissecting channels lined by atypical, multilayered endothelial cells and an additional high-grade epithelial component. RHE is a pure pattern and lacks other vascular components [56,60]. EHE is also a pure pattern lacking another vascular component pattern. Spindle cell hemangiomas show predominant spindle cell morphology with cavernous and epithelioid patterns accompanying thrombi and phleboliths [55,57,58].

#### 3.6.8. Current Literature on Biological Behavior 

CHE’s are currently defined as intermediate neoplasm due to their potential for local recurrence and rare metastasis. Current studies suggest that local recurrence is estimated at 57%, ranging from 18 months to ten years from the original excision [59]. The current literature does not distinguish any difference in biologic behavior and prognosis among cases with various combinations of the above histologic patterns, including lesions with an angiosarcomatous component [55,56,57]. Due to its classification as an indeterminate neoplasm, wide local surgical excision is recommended as a first-line treatment protocol [58,60,61]. Overall, CHE’s show relatively high rates of recurrence, warranting close follow-up.

Patients with metastases or multiple tumors require more aggressive treatment strategies. For example, Sakamoto et al. demonstrated that patients with multiple tumors of the foot and sole were treated with extensive resection and radiotherapy. In addition, these patients were followed for 2.5 years through positron emission tomography with 2-deoxy-2-[fluorine-18] fluoro-D-glucose without recurrence or metastasis [62].

## 4. Conclusions

Cutaneous vascular neoplasms of uncertain biological behavior remain a challenging area for both pathologists and clinicians prompted by their infrequent occurrence in daily practice and overall ambiguous guidelines for treatment and follow-up. However, as more cases continue to be reported in the literature, the fund of knowledge regarding the most successful treatment and follow-up strategies for patients continues to emerge. This paper aims to provide a comprehensive overview of each entity with acknowledgments of the novel molecular alterations and treatment strategies (Table 1).

Overall, the review of available literature supports the notion that most superficial tumors amenable to wide local excision have an excellent prognosis with a low probability of recurrence. In contrast, deeper and more extensively infiltrative tumors not amenable to a conservative excision continue to present a conundrum. A review of the current literature has demonstrated several studies in which conservative radiotherapy [63] or the use of targeted drugs (e.g., Telatnib and Sirolimus) [7,11] provides excellent outcomes for patients, demonstrated by high regression rates.

Lastly, follow-up guidelines for patients should be determined by the specific treatment method utilized and the original tumor identity. Based upon the review of the literature, patients with tumors amenable to complete wide local excision can undergo longer time intervals between follow-up as the overwhelming majority of patients had no recurrence. Overall, the literature suggests an initial follow-up for such patients at six months’ post-surgical excision to ensure appropriate healing and no recurrence. After an initial follow-up, clinicians may only see a patient when a specific concern arises. For patients with deep, infiltrative tumors on systematic therapies, a shorter and more close follow-up period remains more appropriate. Initially, regularly scheduled imagining modalities of the mass should be employed to assess the degree of response to therapy. Patients with large, infiltrative tumors should be seen monthly until the documentation of tumor regression. Once regression has been achieved, a longer interval follow-up time would be reasonable. Once remission is achieved, annual follow-up or longer time intervals between visits can be planned. Patients with documented metastases, multifocal tumors, or local recurrence should undergo strict follow-up and monitoring. 

## Figures and Tables

**Table 1:** Summary of cutaneous vascular neoplasms with diagnostic features and treatment strategies.

Neoplasm	Histological Appearance	IHC	Molecular	Treatment Strategies
KHE	-Spindled endothelial cells with slit-like spaces and ectatic vessels in a collagenous stroma -Deep dermal involvement with extension into the subcutaneous tissue -Dilated lymphatics -Rare mitoses -Minimal atypia -Rare necrosis	Positive for: CD31, CD34, FLI-1, EGR, D2-40 Negative: GLUT1, HHV8	*Prox1* *GNA14*	-Superficial tumors are usually cured by wide excision -Deep tumors not amendable to excision are best treated with Sirolimus-containing therapies with excellent chances of regression -Transarterial embolization in patients with KMP
EPA	-Dermal based vascular proliferation that may involve subcutis -Large cuboidal endothelial cells with hobnailing -Cytoplasmic vacuolization -Rare mitosis -Surrounding lymphocytic infiltrate and sclerotic collagen	CD31+ Positive for: CD34, ERG, FLI-1, D2-40, VEGFR-3	None currently documented	-Most tumors are treated with complete wide local excision -Local radiation therapy for large masses with deep invasion or positive resection margins
PHE	-Infiltrative borders, ill-defined Nodules/sheets/fascicles of plump spindle/epithelioid cells with abundant eosinophilic cytoplasm -Rhabdomyoblastic-like morphology -Fibrous/desmoplastic stroma -Prominent neutrophils in some cases -Absence of well-formed vessels -Rare necrosis	Positive for: AE1/AE3, ERG, FLI-1, CD31 (50%), FOSB Negative for: CD34, Desmin, Myogenin, CAMTA1	*SERPINE1*- *FOSB*	-Superficial tumors treated with complete wide local excision -Telatinib for patients with multifocal tumors or tumors not amenable to resection
RHE	-Elongated, arborizing vessels resembling rete testis -Papillary intraluminal projections -Hobnail endothelial cells -Significant histological overlap with EPA	Positive for: CD31, CD34, ERG, FLI-1, Negative for: D2-40, VEGFR-3	None currently documented	-Complete wide local excision is preferred -Chemoradiation with cisplatin and radiotherapy for tumors not amenable to resection
EHE	-Infiltrative Epithelioid endothelial cells showing intracytoplasmic vacuoles (blister cells) -Myxohyaline stroma -Rare mitosis -Absence of well-formed vascular channels -Metaplastic calcification or ossification -Rare necrosis	Positive for: CD31, CD34, ERG, FLI-1, TTFE3, CAMTA1	*WWRT1- CAMTA1*	-Complete wide local excision is preferred -Mohs surgery has demonstrated efficacy for sensitive anatomic locations
CHE	-Mixture of various vasiform patterns in one single tumor -RHE pattern is the most common -EHE second most common pattern	Positive for: CD31, CD34, ERG, FLI-1	Consistent with histologic patterns present -*PTBP1*- *MAML2* -*EPC1*- *PHC2*	-Complete wide local excision is the preferred treatment -Multiple tumors require aggressive resection and radiation therapy

Abbreviations: CHE: composite hemangioendothelioma; EHE: epithelioid hemangioendothelioma; EPA: endovascular papillary angioendothelioma; KHE: kaposiform hemangioendothelioma; KMP: Kasabach-Merritt phenomenon; PHE: pseudomyogenic hemangioendothelioma; RHE: retiform hemangioendothelioma.

## Data Availability

No new data were created or analyzed in this study. Data sharing is not applicable to this article.
